# Adolescent pregnancy: An important issue for paediatricians and primary care providers—A position paper from the European academy of paediatrics

**DOI:** 10.3389/fped.2023.1119500

**Published:** 2023-02-07

**Authors:** Miguel Vieira Martins, Nora Karara, Lukasz Dembiński, Martine Jacot-Guillarmod, Artur Mazur, Adamos Hadjipanayis, Pierre-André Michaud

**Affiliations:** ^1^ Young European Academy of Paediatrics, Brussels, Belgium; ^2^ Portuguese Society of Pediatrics/Sociedade Portuguesa de Pediatria-SPP, Lisbon, Portugal; ^3^ Child and Youth Public Health Service, Berlin, Germany; ^4^ European Academy of Paediatrics, Brussels, Belgium; ^5^ Department of Pediatric Gastroenterology and Nutrition, Medical University of Warsaw, Warsaw, Poland; ^6^ Department Women-Mother-Child, Lausanne University Hospital (CHUV), Lausanne, Switzerland; ^7^ Faculty of Medicine, University of Rzeszów, Rzeszów, Poland; ^8^ Medical School, European University of Cyprus, Nicosia, Cyprus; ^9^ Faculty of Biology & Medicine, Lausanne University, Switzerland

**Keywords:** adolescence, pregnancy, sexual reproductive health, contraception, healthcare policies

## Abstract

Adolescent pregnancy and childbearing, remain a widespread health-related problem with potential short and long-term consequences. Comprehensive social, economic, environmental, structural, and cultural factors heavily impact on adolescents' sexual and reproductive health and early pregnancy. Health professionals can play a pivotal role in the prevention of unplanned pregnancy. Improved access to family planning, sexuality education in schools, community-based interventions, and policies contribute greatly to reduce the risk of adolescent pregnancy and the adoption of respectful and responsible sexual behaviour. Additionally, health care professionals can support pregnant adolescents in making decisions under these circumstances and provide adequate health care. This review highlights actions that can guide healthcare professionals in empowering young adolescents to become more aware and capable of making informed decisions about their sexual life, health, and future.

## 1. Introduction

Adolescence is one of the most formative phases of human development with selective physical, cognitive, emotional, social and sexual maturation. It is also the time of seeking the balance between risky and protective behaviour, personality education, consolidation of social roles and lifestyle choices (1, 2). A major issue during adolescence is the emergence of the sexual and reproductive health (SRH), with potential health-compromising consequences such as unintended pregnancy, which remains a public health concern worldwide among girls aged 10–19 years. According to data from the United Nations from 2015 to 2020, in European countries like the United Kingdom or France, 12 and 9 in every 1,000 women under 18 years had a delivery, respectively. These birth rates do not compare with those of low-income countries. An estimated 21 million girls aged 15 to 19 years in developing regions become pregnant every year, and approximately 12 million give birth (3). Although the issue of adolescent pregnancy represents a major issue in low-and-middle income countries, it is still a matter of concern in many European countries.

Several recent publications have assessed the trends in pregnancy rate observed in high-, middle- and low-income countries: in all part of the world, the percentage of girls aged 10 to 19 becoming pregnant has decreased (4, 5). According to Part et al. (6), teenage pregnancy rates in European countries have declined since 2,001 overall. This trend is however not similar across countries, as estimates from the WHO in 2021 point to higher rates of pregnancy in women aged 15–19 years in Eastern Europe (16.7 births per 1,000 women), when compared with Northern (8.29/1000), Western (7.35/1000) and Southern Europe (6.64/1000) (7) On a closer look, the UK has registered one of the most important drop with a reduced rate of adolescent pregnancy by 51% from 1998 to 2014 (8), due to its “*Ten-year teenage pregnancy strategy for England”* that improved access to contraception, and increased the reach to young people and parents. On another note Switzerland, since many years, exhibits the lowest rate of the world (4, 9) in light of long-standing sexual education programs and access to family planning clinics for adolescents. Of note, estimates of pregnancy may be underestimated, as is the case with abortions in adolescents, which are unavailable or incomplete in more than one-third of EU countries. Interestingly, reported teenage pregnancy rates seem generally lower for European countries in which parental consent for abortion is not required and in which SRH services are available in all areas, including not rarely a subsidized access to contraception for minors (6). This paper gives an overview on the issue of adolescent pregnancy, particularly in Europe, focusing on predisposing and preventing factors, as well as the promotion of safe and conscious options regarding unexpected pregnancy.

## 2. The Various facets of adolescent pregnancy

### 2.1. Factors predisposing to adolescent pregnancy

Multiple predisposing factors have been associated with adolescent pregnancy (1), some of them being linked with personal vulnerability, family context, lack of information in the area of SRH, poor access to contraception, environmental violence, tight socio-economic context, armed conflicts, religion and cultural background [e.g., early marriage (10)]. Adolescents, especially under the age of 16 or 17 years lack a long-term vision of the consequences of their behaviour and are driven by experimentation and emotions (11, 12). Therefore, although they cognitively know about contraception and the risk of pregnancy, they may chose to ignore these aspects of their behaviour as a kind of denial (“it cannot happen to me”). This explains why, even in high income countries with a good coverage of health care needs, some adolescents find themselves pregnant. However, in most circumstances, adolescent pregnancy results from lack of information and poor access to contraception (1, 13–15). Adolescents who become pregnant often exhibit risky behaviour such as not using contraception consistently or using of less-effective contraceptive methods, and engaging in sexual activity under the influence of alcohol, stimulants or drugs (9). In addition, adolescents facing situations of vulnerability, such as victimization, violence and war, or extreme socio economic deprivation are at higher risk of finding themselves pregnant. For instance, in all countries of the world, young people from migrant or refugee backgrounds may face obstacles in getting SRH counselling, due to ignorance, difficult communication and inequitable access to healthcare.

Along the same line, adverse circumstances may encourage teenagers to engage in childbearing as a way to keep some future prospect, especially when the family and the community environment reinforce such beliefs (16).

### 2.2. The role of male partners in adolescent pregnancy

Although studies in preventing pregnancy in adolescents have been known to focus more on females, the role of young men is also very relevant. The World Health Organization (WHO) proposes to develop adolescent pregnancy prevention efforts that are focused on both young men and women (17).Young men consistently demonstrate having low knowledge of contraception and sexual health options, when compared to young women (17). Notably, they are more aware of male-dependent methods of contraception, such as condoms (or withdrawal), but lack knowledge on hormonal methods, like short or long-acting reversible contraception. Moreover, young men also fall short in knowledge of hormonal emergency contraception. Limited knowledge of these subjects reduces confidence and ability to discuss sexual health with their female partners (17). These facts enlighten the importance of including young males in sexual health education, and encouraging the recognition of their responsibility toward the prevention of a pregnancy.

### 2.3. Medical and psychosocial consequences of adolescent pregnancy

In European high-and-middle income countries, many pregnant adolescents, especially under 18 years engaged in a professional career (high-school, apprenticeship) will decide to perform an abortion, and the rates of children born to adolescents have gone down in many European countries (18). It is difficult to obtain valid figures in this respect, but suffice is to say that adolescent abortions represent between 10 and 20 percent of all legal abortions registered in European countries (19). The rate of abortions among 15 to 19 year-old adolescents varies 6 per 1,000 females (Germany) and ∼20 (Estonia, Hungary) (19). It should be kept in mind however that there are still important differences between European countries in terms of access to abortion, which largely depend on their national health systems and legal framework (6). Despite the fact that most legislations guarantee some access to such intervention, it remains a fragile right in some European countries (20). Moreover, it should be stressed that, if performed by expert health professionals, abortion is safe and does not lead to unfavourable health consequences (21).

The medical consequence of adolescent pregnancy is highly dependent on the context of health care delivery. In high income countries, with an adequate obstetrical follow-up, pregnant adolescents do not face more complications than older adults and indeed seem to do better (22, 23). However, pregnant adolescents, especially those engaging in risky behaviour, who are victims of sexual violence or who do not benefit from adequate sexual education are at risk for health threatening situations, not only of infectious nature, like urinary tract infections, chorioamnionitis, pyelonephritis and sexually transmitted infections (STIs), but also of other conditions such as eclampsia, preeclampsia and anaemia (24, 25). In addition, depending on the quality of the obstetrical follow-up, pregnant adolescents are at higher risk of foetal and neonatal complications such as lower birth weight, growth restriction, infection and sudden infant death syndrome (25, 26). Preterm birth in these girls is also worrisome since the risk of early delivery is higher than in older mothers (27). This is particularly true in repeated pregnancies within 2 years of the first one, where the risk of preterm birth increases almost twice-fold (27, 28).

A study tackling Swedish statistics from the last century suggests that teenage mothers, independent of socio-economic background, face an increased risk of premature death (29).

Adolescents living with a chronic condition face some further sexual health burden: on one hand, hormonal contraceptives can affect the course of disease and interact with medications (30). On the other hand, the occurrence of a pregnancy may impact negatively on the progress of the diseases such as congenital heart disease or pro-thrombotic conditions, among others. In addition, under some circumstances, mental health disorders may foster risky sexual behaviour (30).

Depending on the family and social environment as well as the support provided by health and social professionals, adolescent mothers are at risk for mental health problems, both during and after the pregnancy. Postpartum depression is a worrying concern in teenage mothers. For instance, an American prospective study indicated that more than half (57%) of these mothers experience moderate to severe depressive symptoms within the first 4 years postpartum (31). A recent Canadian study pointed out the fact that around 50 per 1,000 adolescents with a major mental illness become pregnant, as compared to 15 per 10,000 adolescents from the general population (32). Thus, mental health problems can constitute both a reason for and a consequence of pregnancy, even though this is more common among adolescents living in an unfavourable psychosocial environment.

In low-income countries, motherhood is sadly considered as a valuable and imperative social status, which makes childbearing an inevitable and even a positive option (1, 25). However, in many European countries this is not the case. In some circumstances, pregnant adolescents develop low self-esteem, endure stigma from their network or even bullying, rejection or violence by partners or family members (33). Furthermore, one of the most distressful potential consequence of pregnancy is a break in the educational track, with long-term impact on the adolescent professional life. These girls often do not complete their education and are less qualified to receive welfare benefits, or are not able to live independently. This is true in any country, wealthy or less wealthy, and is highly dependent on the socio-economic level of the family and the availability of support from the community (e.g., special school program for pregnant adolescents) (1, 25, 34, 35).

## 3. Discussion

### 3.1. A role for paediatricians and primary care providers

In most European countries, the care of pregnant adolescents is in the hands of gynaecologists, but there is a role for primary care providers to advice adolescents as well. This topic still remains a difficult health care task to perform for many primary care providers, and in some instances a real taboo. This keeps adolescents from disclosing active sexual relationship and of seeking advice in the area of contraception and STIs or suspected pregnancy. Studies show that Primary Care Physicians and Paediatricians' offices alone are not a teenagers' preferred option when it comes to discussing their sexual behaviours (36, 37). A recent survey conducted in all EU countries and tackling the existence of formal recommendations in the area of SRH counselling for adolescents concludes that “the provision and availability of youth-friendly SRH care are far from optimal in around half of the surveyed countries” (38). Moreover, the training of primary care doctors in the area of adolescent SRH is far from optimal: in only 22 countries out of 39 do paediatricians receive a formal education in the area of adolescent SRH, the number being even lower as far as family practitioners are concerned (17 out of the 39 countries) (39).

The WHO and other authors have developed several recommendation as how to make health care to adolescent more friendly (37, 40, 41). Key elements that impact on the quality and effectiveness of service provisions are, among others, easy access to health services, including SRH; confidential, respectful empathetic care; communication and counselling skills; easy link with specialized colleagues and the community. Specifically, as far as SRH is concerned, the role of paediatricians and primary care professionals should cover (see Table 1) both preventive and care issues. They should ideally integrate contraceptive care, sexual health awareness and counselling about birth and abortion options, in their daily encounters with adolescents. In fact, tackling these issue within the global appraisal of any adolescent's lifestyle is a key task of practitioners, including the use of the well-known HEADDSSS acronym (42, 43). A detailed description of how to handle contraception issues and adolescent pregnancy can be found in a very recently issued “Pocket book of primary health care for children and adolescents”, which is widely available online (44).

**Table 1:** Role of Paediatricians and Primary Care Providers in the area of adolescent SRH (this applies to both girls and boys).

1. Provide confidentiality, establish a trustful empathetic relationship
2. Include in any consultation with an adolescent, as far as possible, a review of lifestyle habits, including attitudes towards gender issues, attitudes vis-à-vis sexual life, specific knowledge in the area of contraception and STIs
3. Review in a non judgemental way, sexual experiences and behaviour
4. Systematically provide information on emergency contraception (even to non sexually active adolescents): Levonorgestrel-only up to 72 h after unprotected sexual intercourse, 1.5 mg as a single dose or two 0.75 mg tablets
5. Explore the use of contraception and the various related options. If adolescent sexually active, provide counselling. Refer for oral contraception if appropriate (or alternatively, primary care professionals can provide oral contraception themselves if adequately educated in the field)
6. If adolescent girl sexually active, inquire about date of last menstruations. If any doubt, proceed with a pregnancy test
7. If pregnancy test positive, provide support to the adolescent, explore representations and discuss options, then refer to gynaecologist. Depending on the legal frame and the adolescent's competence, decide whether and how to involve partner and the family/caregivers

Many professionals tend to think that adolescents living with a chronic condition engage in exploratory/risk behaviour later or less frequently than their healthy mates. This notion is actually a wrong belief, as various surveys show that it is actually the reverse which is true (45). In other terms, exploring the various aspects of sexual life as described in Table 1 constitutes a full part of the health care of young patients with any kind of chronic condition.

For an adolescent, discovering a positive test that confirms a pregnancy comes most of the time as a shock, especially if unexpected. In this situation, the young pregnant girl needs a judgement-free, neutral and supportive information about pregnancy, maternal and paediatric health. Birth options and available alternatives including pursuing the pregnancy with the support of the family and of available social services, abortion, or in some instances adoption plans should also be available. It is important to mention that providing confidential counselling and advice on the availability of legal abortion has an effect on access to affordable and safe procedures, thus reducing the odds of short and long-term medical complications (1). In most European countries, both an abortion procedure and the follow-up of the pregnancy will be in the hand of gynaecologist. Thus, issues such as diagnosis of anaemia and other nutritional deficiencies, screening for STIs and assessment of immunologic status are often not part of the paediatrician's mission. Their main role is to identify pregnancies at-risk and make early referrals for adequate follow-up, which will secure a safe progress of the pregnancy (22), or access to safe abortion (46). Ensuring competent follow-up care, including information and access to contraception after birth, pregnancy testing and post-partum counselling has proven an effective way to support adolescents in this critical time (8).

Families heavily involved in helping and supporting their children who are soon-to-be parents and facilitating counselling sessions could have an important impact in creating a safe network for the young mother and child. Information early on about legislation regarding parental rights and duties such as custody agreements, child support payments and governmental support are essential steps towards autonomy as well.

### 3.2. Prevention of adolescent pregnancy

The prevention of pregnancy, and more globally the prevention of STIs and the promotion of a healthy sexual life has prompted many initiatives in European countries, as well as in the rest of the world (13). They can be broadly classified as three main approaches, the one of sexuality education (mainly school-based), the one of community interventions and the one of policies affecting the adolescents' access to information, to contraception and to SRH care.

#### 3.2.1. Sexuality education

Sexuality education initiatives have been the subject of numerous assessment since around 40 years (47) and then ongoing (48, 49). Most of the time, they aim at decreasing unplanned pregnancy as well as STIs—including HIV—but also to improve the quality of sexual life of young people. According to several recent publications (5, 14, 50), they do not, contrary to a widely spread belief, increase the rate of adolescents becoming sexually active, and contribute to the adoption of responsible and respectful sexual exchanges and experiences. According to Ketting (15), 21 out of 25 European countries have adopted a legal framework for sexuality education. In addition, in many Western and Central European countries, sexuality education in schools is often comprehensive and fully integrated into broader teaching subjects, such as citizenship, life skills or health education initiatives.

#### 3.2.2. Community interventions

Diverse underserved individuals and communities need special approaches to ensure them from getting the information and care they need, sometimes mediated by community leaders (1, 2, 13). Providing helplines *via* chat or phone specifically for teenagers can reduce hesitancy to seek help due to anonymity. Campaigns on a national level should address teenagers *via* their “natural habitat” social media (*TikTok* and *Instagram* are presently most popular) but also encourage parents to talk to their children about sex to demystify the issue. Migrant adolescents are often facing a situation of special vulnerability, as they lack information in the field of SRH and access to effective contraception. In these cases, special outreach and culturally appropriate actions are needed to reach them in their community. There are now scientific evidence that large scale country-based prevention program, involving actively young people can be effective and cost-saving (51).

#### 3.2.3. Policies

Public health administrations as well as various non-governmental organizations (NGOs) can promote initiatives that can impact on adolescents' sexual life and unplanned pregnancy. One of Europe's recent success story regarding government intervention is the UK, which was able to reduce rates of adolescent pregnancy by 51% (from 1998 to 2014) with a national strategy targeting teenagers and their parents through communications campaigns, providing funding for improved sex and relationship education (i.e., in schools) and access to effective contraception (8). Several European countries offer free contraception such as oral combined contraception to minors, including Portugal, Slovenia (for the insured), Belgium, the Netherlands and France. Other countries such as Germany and Spain subsidize contraceptives, whereas in many countries minors have to pay for contraception themselves, making this an important barrier to adherence (6). Furthermore, regulatory hurdles, such as parental consent potentially restrict access to both contraception and medical procedures like abortions (25). As follows, the latter should be considered to help create a safer health environment for adolescents, including securing confidential care (52).

### 3.3. Recommendations of the European academy of paediatrics (EAP)

The EAP's mission is to foster all interventions which ensure the welfare of adolescent mothers and their children at different levels. In this respect, the following recommendations emphasize the the importance of the issue.

**For International and National State, Health and Education Authorities and NGOs:**

• Advocating for free access to contraception for adolescents
• Training staff to convey accurate sex education and family planning in schools
• Providing open and easy access to youth-friendly health care to young women and their partners and facilitate their access to family planning counselling
• Liberalizing free access to safe abortion
• Advocating for adolescent mothers and supporting them by creating opportunities in employment, education and training

**For Health care Providers:**

• Acquiring and maintaining knowledge as well as adequate attitude and communication skills in counselling and delivering health care related to SRH among adolescents, both girls and boys, emphasizing the issue of confidentiality
• Advocating for an easy access to youth-friendly health care, including so-called family planning.
• Developing skills in supporting pregnant adolescents throughout their pregnancy; providing pregnant adolescents information around choices regarding pregnancy, including options such as continuing the pregnancy (and raising the child), considering abortion or giving the child to adoption.
• Delivering the relevant information in a basic, accurate and developmentally appropriate manner, involving the patient in the decision-making process, and respecting the patient's cultural and spiritual background and values
• As far as possible, implicating male partner and parents/caregivers in all decisions.
• Assisting the pregnant adolescent in reaching out to community resources that will support her path throughout and after the pregnancy if the pregnancy is kept.

## Data Availability

The original contributions presented in the study are included in the article/Supplementary Material, further inquiries can be directed to the corresponding author/s.
